# Iatrogenic delirium on symptom-triggered alcohol withdrawal protocol

**DOI:** 10.1108/MIJ-02-2020-0002

**Published:** 2020-04-01

**Authors:** Andrew Chunkil Park, Leigh Goodrich, Bobak Hedayati, Ralph Albert, Kyle Dornhofer, Erin Danielle Knox

**Affiliations:** Department of Psychiatry and Human Behavior, UC Irvine Healthcare, Orange, California, USA; Department of Medicine, University of California Irvine, Irvine, California, USA; Department of Psychiatry and Human Behavior, UC Irvine Healthcare, Orange, California, USA

**Keywords:** Alcohol withdrawal, Iatrogenic delirium, Delirium, Benzodiazepines

## Abstract

**Purpose:**

The purpose of this paper is to illustrate delirium as a possible consequence of the application of symptom-triggered therapy for alcohol withdrawal and to explore alternative treatment modalities. In the management of alcohol withdrawal syndrome, symptom-triggered therapy directs nursing staff to regularly assess patients using standardized instruments, such as the Clinical Institute for Withdrawal Assessment of Alcohol, Revised (CIWA-Ar), and administer benzodiazepines at symptom severity thresholds. Symptom-triggered therapy has been shown to lower total benzodiazepine dosage and treatment duration relative to fixed dosage tapers (Daeppen *et al.*, 2002). However, CIWA-Ar has important limitations. Because of its reliance on patient reporting, it is inappropriate for nonverbal patients, non-English speakers (in the absence of readily available translators) and patients in confusional states including delirium and psychosis. Importantly, it also relies on the appropriate selection of patients and considering alternate etiologies for signs and symptoms also associated with alcohol withdrawal.

**Design/methodology/approach:**

The authors report a case of a 47-year-old male admitted for cardiac arrest because of benzodiazepine and alcohol overdose who developed worsening delirium on CIWA-Ar protocol.

**Findings:**

While symptom-triggered therapy through instruments such as the CIWA-Ar protocol has shown to lower total benzodiazepine dosage and treatment duration in patients in alcohol withdrawal, over-reliance on such tools may also lead providers to overlook other causes of delirium.

**Originality/value:**

This case illustrates the necessity for providers to consider using other available assessment and treatment options including objective alcohol withdrawal scales, fixed benzodiazepine dosage tapers and even antiepileptic medications in select patients.

## Report

The patient, a 47-year-old Caucasian male, was brought to a local emergency department (ED) by emergency medical services (EMS) after being found down in a local park with bradycardia and subsequent pulselessness. EMS were able to achieve return of spontaneous circulation in the field after cardiopulmonary resuscitation and administration of epinephrine 1 mg IV.

On arrival to the ED, the patient was obtunded with Glasgow Coma Scale score of 3 and was subsequently intubated. He was administered naloxone 0.4 mg IV and no response was elicited; flumazenil 0.2 mg IV was then administered, which elicited spontaneous right lower extremity movement. At admission, his blood alcohol concentration was 368 mg/dL and a urine drug screen was positive for benzodiazepines. Of note, an empty alprazolam container was found amongst his personal possessions. His past psychiatric history was notable for schizoaffective disorder, depressive type, benzodiazepine use disorder and alcohol use disorder with multiple previous hospitalizations for reasons including suicide attempts by overdose. At a later interview, patient reported compliance with home quetiapine, though he had a history of noncompliance according to family members. The patient reported alprazolam use only and denied long-acting benzodiazepine use including clonazepam and diazepam, though this information was not initially available to providers.

The patient was admitted to the cardiac intensive care unit (ICU) from the ED for post-cardiac arrest care. Admission laboratory studies were notable for liver function tests within normal limits. The patient was observed to become progressively agitated on hospital day 2, with dexmedetomidine, propofol and midazolam infusions titrated to target Richmond Agitation-Sedation Scale (RASS) scores of 0 to −2. Notably, the patient was administered midazolam 41 mg IV total on hospital day 5. The patient was extubated on hospital day 5, and Clinical Institute for Withdrawal Assessment of Alcohol, Revised (CIWA-Ar) was started, at least in part, because it was unknown whether the patient was experiencing withdrawal symptoms from long-acting benzodiazepines. Elevated CIWA-Ar scores recorded by nursing staff from hospital days 6 to 9 triggered increasing benzodiazepine administration, peaking with 36 lorazepam milligram-equivalents on hospital day 8. His vital signs trended upwards following extubation with a blood pressure range of 90-159/52-81 mm Hg, heart rate range of 94-141 beats/min and respiratory rate range of 12-41 breaths/min.

**Figure 1:**
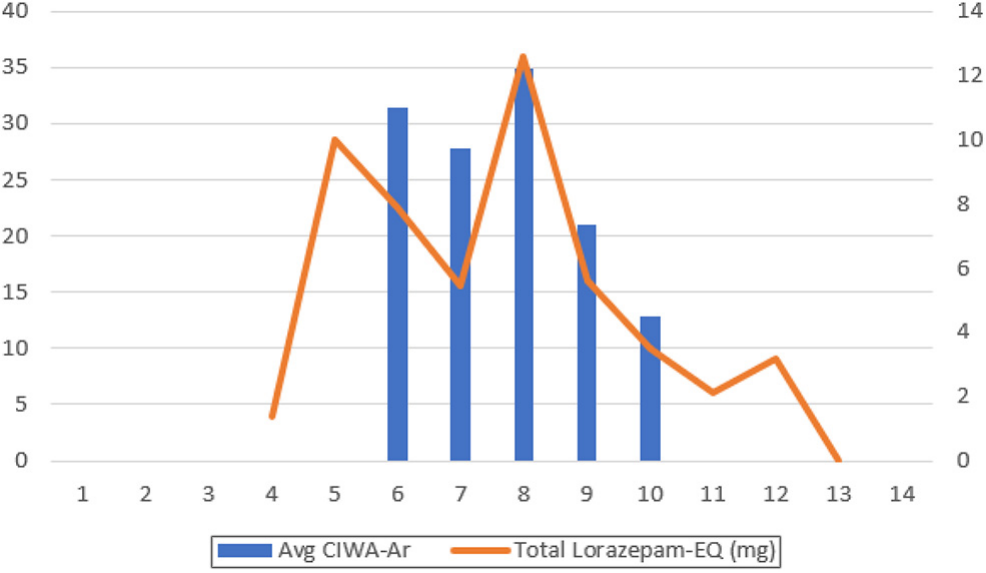
Average CIWA-Ar scores vs total lorazepam milligram-equivalents

The psychiatry consult and liaison service were consulted for management of the patient’s agitation on hospital day 8. The patient was observed to demonstrate waxing and waning attention, orientation and agitation, with serial Confusion Assessment Method for the ICU (CAM-ICU) screens positive for delirium (Inouye *et al.*, 1990). During non-agitated periods, the patient reported auditory hallucinations and demonstrated mild thought process disorganization consistent with psychosis. Given these findings, the patient was started on a fixed dosage benzodiazepine taper and haloperidol as needed for agitation and was uptitrated on risperidone started at a low dose by the primary team for schizoaffective disorder. After the patient denied long-acting benzodiazepine use, lorazepam milligram-equivalents of 16 , 10 , 6 and 9 mg were administered on hospital days 9, 10, 11 and 12, respectively. Note, a benzodiazepine as needed dose was administered on hospital day 12 in addition to the planned taper. Risperidone was uptitrated to 2 mg twice daily by hospital day 12. The patient’s waxing and waning attention, orientation and agitation were resolved, and multiple CAM-ICU screens were negative. The patient’s thought process disorganization improved, and he did not endorse auditory or visual hallucinations or obvious delusions. He was assessed to be at low risk for acute self-harm, as he denied suicidality, contracted for safety and demonstrated future orientation by articulating plans to re-enroll in Social Security Income and to return to his previous living arrangements. The patient was then discharged with close outpatient follow-up (Figure 1).

Graphical representation of total lorazepam milligram-equivalents administered per CIWA-Ar protocol on hospital days 6 to 8 and on a fixed dosage taper from hospital days 9 to 13.

## Discussion

The prevalence of delirium has been reported to be as high as 80 per cent in critical care patients (Girard *et al.*, 2008). The patient in this case was at high risk for developing delirium because of his history of toxic ingestion as well as intubation, associated pain and possible anoxic brain injury while admitted to the ICU. The onset of delirium in this patient preceded its diagnosis and likely occurred while he was being intubated because of his high RASS scores. The continuation of CIWA-Ar after peak alcohol withdrawal period in this case may have been appropriate given his unreliable history regarding long-acting benzodiazepine use. However, benzodiazepines have not been shown to be an effective treatment of non-alcohol-related delirium and have, in fact, been demonstrated to be an independent risk factor in ICU patients (Lonergan *et al.*, 2009; Pandharipande *et al.*, 2006; Breitbart *et al.*, 2005). The worsening of delirium symptomatology with escalating benzodiazepine dosages per CIWA-Ar protocol and its rapid resolution with the fixed benzodiazepine dosage taper support benzodiazepines as a possible precipitant and likely perpetuating factor.

While symptom-triggered therapy through instruments such as the CIWA-Ar protocol have shown to lower total benzodiazepine dosage and treatment duration in patients in alcohol withdrawal, over-reliance on such tools may also lead providers to overlook other causes of delirium (Daeppen *et al.*, 2002; Hecksel *et al.*, 2008). CIWA-Ar is further limited by its highly subjective nature, its susceptibility to inappropriate patient selection and barriers to application where there are communication issues with the patient because of medical or language barriers (Knight and Lappalainen, 2017). Finally, CIWA-Ar has not been validated for patients in medical and surgical ICUs (Bostwick and Lapid, 2004).

While the patient’s presentation may be explained by an acutely decompensated psychotic episode alone, it is more likely that the patient was experiencing delirium in addition to psychosis. The patient was observed to be confused with poor reality testing during non-agitated periods, which could be explained by both conditions. However, his agitation and confusional state had a fluctuating course more consistent with delirium. The patient also had a known history of medication noncompliance and multiple acutely decompensated psychotic episodes requiring hospitalization. Misattribution of delirium is common in patients with psychotic and depressive disorders, both disorders of which the patient had a known history, which may have delayed its diagnosis here (Ely *et al.*, 2004).

## Conclusion

We have reported a case of worsening delirium developed on the CIWA-Ar protocol. This case illustrates the necessity for providers to consider using other available assessment and treatment options including objective alcohol withdrawal scales, fixed benzodiazepine dosage tapers and even antiepileptic medications in select patients. An effective systems-based approach may include setting time limits on CIWA-Ar orders within the electronic medical records to allow for reassessment of its appropriate use.
